# Invasive Ant Detection: Evaluating Honeybee Learning and Discrimination Abilities for Detecting *Solenopsis invicta* Odor

**DOI:** 10.3390/insects15100808

**Published:** 2024-10-15

**Authors:** Suwimol Chinkangsadarn, Lekhnath Kafle

**Affiliations:** 1 Department of Tropical Agriculture and International Cooperation, National Pingtung University of Science and Technology, Pingtung 912, Taiwan; chinkangsadarn_s@su.ac.th; 2 Faculty of Animal Sciences and Agricultural Technology, Silpakorn University, Phetchaburi 76120, Thailand

**Keywords:** bio-detection, latent inhibition, odor generalization, proboscis extension response, red imported fire ant, surveillance methods

## Abstract

Invasive red imported fire ants (*Solenopsis invicta*) create a serious threat to public safety, agriculture, biodiversity, and the local economy, necessitating early detection and surveillance, which are currently time-consuming and dependent on the inspector’s expertise. This study marks an initial investigation into the potential of honeybees (*Apis mellifera*) to detect and discriminate the odor of *S. invicta* through the olfactory conditioning of proboscis extension responses. Deceased *S. invicta* were used as conditioned stimuli to ensure relevance to non-infested areas. The results showed that the bees rapidly learned to respond to deceased ant odors, with response levels significantly increasing at higher odor intensities. Bees exhibited generalization across the odors of 25 minor workers, 21 median workers, 1 major worker, and 1 female alate. When conditioned with deceased ant odors, bees effectively recognized live ants, particularly when trained on a single minor worker. Discrimination abilities varied by species and were higher when *S. invicta* was paired with *Polyrhachis dives* and *Nylanderia yaeyamensis*, and lower with *S. geminata*, *Pheidole rabo*, and *Pheidole fervens*. Notably, discrimination improved significantly with the application of latent inhibition. These findings suggest that trained honeybees have the potential to detect *S. invicta*. Further refinement of this approach could enhance its effectiveness for detection and surveillance.

## Simple Summary

This study explores the potential of honeybees to detect and differentiate the odor of invasive red imported fire ants (*Solenopsis invicta*) using olfactory conditioning. By training bees with deceased ants, the results showed that bees quickly learned to recognize ant odors, with stronger responses to higher odor intensities. Bees generalized well across different worker castes and female alates and could recognize live ants after being conditioned with deceased ones. Discrimination varied by species but improved with a latent inhibition procedure. The findings suggest that honeybees may serve as a valuable tool for *S. invicta* detection and surveillance.

## 1. Introduction

The red imported fire ant, *Solenopsis invicta*, is widely known as one of the most detrimental invasive ant species globally, with far-reaching consequences for public safety, agriculture, biodiversity, and the local economy [1]. Its sting can cause recurring pain, allergic reactions, severe cellulitis, and even anaphylactic shock [2,3,4,5,6]. It is native to South America and was introduced to the United States in the 1930s. Since then, this fire ant has rapidly spread to over 20 countries and territories, including the Caribbean, New Zealand, Australia, Hong Kong, Macau, China, Taiwan, South Korea, and Japan [2,7,8,9,10,11]. The primary means of dispersal is through international trade and transportation, particularly cargo ships [7,12].

To control the spread and mitigate the ecological and economic impacts, implementing early detection and surveillance is crucial [9,13]. However, traditional methods have several limitations, particularly in identifying *S. invicta* among collected specimens. It is labor-intensive, time-consuming, and heavily reliant on inspector’s expertise. Challenges include the presence of morphologically similar species, variability in ant morphology within the species, and the capture of multiple ant species in traps, which complicates accurate identification and population assessment [14].

To address these limitations, advanced techniques, such as lateral-flow immunoassay [15] and loop-mediated isothermal amplification, have been developed [16]. Despite their effectiveness, these methods are costly and require specialized equipment and trained personnel. In contrast, detection dogs offer a highly sensitive and efficient alternative for locating *S. invicta* mounds and odors [12,17,18]. They provide advantages over other methods in terms of flexibility and real-time results. However, their effectiveness can be influenced by environmental conditions and operational costs.

Honeybees (*Apis mellifera*), endowed with olfactory senses comparable to those of detection dogs, can detect a wide array of substances, including explosive materials [19,20,21], tuberculosis [22], fruit fly-infested oranges [23], and SARS-CoV-2 [24]. Given their sensitivity, honeybees could be considered as an alternative tool for detecting fire ants in situations where other detection methods encounter limitations.

Honeybees can be trained to detect odors using olfactory conditioning of the proboscis extension response (PER) protocol, where they learn to associate a sucrose reward with specific odors [25,26,27]. This training process is particularly brief, typically requiring only a few hours. It is a cost-effective method compared to training dogs and can be conducted in any location where beekeeping is practiced [27,28].

This study aimed to assess the feasibility of utilizing honeybees to detect and discriminate *S. invicta* odors through the olfactory conditioning of PER. The study was carried out to determine the following objectives: (i) the learning performance of honeybees with deceased ant odors; (ii) the response levels of honeybees to varying intensities of deceased ant odors; (iii) generalization of honeybee responses across different deceased ant odors; (iv) the recognition of live ants by honeybees conditioned to deceased ant odors; and (v) the discrimination of ant odors by the conditioned honeybees.

## 2. Materials and Methods

### 2.1. Insects

#### 2.1.1. Red Imported Fire Ant

Red imported fire ants (*S. invicta*) were collected from mounds in Banqiao, New Taipei City, Taiwan. For experiments involving deceased ants, foragers were attracted with potato chip baits placed along their trails for 20–30 min [1]. The ants were euthanized using cold anesthesia; the deceased ants were shipped to National Pingtung University of Science and Technology (NPUST), Pingtung, Taiwan, a non-infested area. These procedures adhered strictly to quarantine regulations allowing for only deceased *S. invicta* specimens to be transported outside the infestation zone [12]. Ant samples were stored at −20 °C and allowed to rest at room temperature for 2 h before use. Experiments with live *S. invicta* were conducted in a designated pest-affected area at National Taiwan University (NTU), with colonies maintained in soil (see Kafle et al. [29] for environmental and feeding details). This research focused on the worker and female alate castes due to their high numbers and frequent encounters during fire ant inspections [11], while male alates and queens are less commonly observed [11,30]. The specimens were categorized based on head width, distinguishing them as female alates (1.4 mm, with wings), major workers (1.21–1.36 mm), median workers (0.93–1.15 mm), and minor workers (0.56–0.66 mm).

#### 2.1.2. Non-Red Imported Fire Ants

Five ant species were selected for the study. The tropical fire ant (*Solenopsis geminata*), an invasive species in Taiwan [31,32], is closely related to, and ecologically similar to, *S. invicta* [32,33]. The study also included ground-dwelling species (*Pheidole fervens* and *Pheidole rabo*) and ground-foraging species (*Polyrhachis dives* and *Nylanderia yaeyamensis*), which are likely to co-exist with *S. invicta* [34,35]. All specimens were collected from various locations within NPUST using potato chip bait, as described previously.

#### 2.1.3. Honeybees

Pollen foragers of Italian honeybees (*Apis mellifera ligustica*) were collected in the morning at the hive entrance, which was temporarily blocked with a wooden stick to slow their return into the hive. Each bee was individually placed in a glass vial with a perforated cap for ventilation. For the experiments with deceased ants, honeybees were collected from an apiary at NPUST (22°38′43.2″ N 120°36′19.8″ E; 67 m altitude) during the period from 2022 to 2024. For the experiments with live ants, honeybees were collected from an apiary at NTU (25°00′39.7″ N 121°32′25.5″ E; 9 m altitude) in March 2024. Individual bees were placed in glass vials and temporarily immobilized on ice before being transferred to harness tubes [36,37,38]. Each bee was then fed a 20% sucrose solution until satiated, followed by a 3 h fasting period before the experiment began.

### 2.2. Experimental Apparatus

In this study, the conditioned stimuli (CS) were odors emitted by ants, delivered using 1 cc disposable plastic syringes with nylon mesh covers as odor cartridges. The unconditioned stimulus (US) was a 50% sucrose solution (*w*/*w*), applied to the bee antenna with a toothpick and then offered to the extended proboscis as a reward.

In the conditioning arena, an individual bee was positioned 2 cm from constant airflow sources (2.5 L/min) with the exhaust behind it [39]. The airflow comprised a primary stream at 2 L/min controlled by an airflow meter, (LZB-4WB, Changzhou Shuanghuan Thermo-Technical Instrument Co., Ltd., Jiangsu, and China) and a secondary stream at 0.5 L/min controlled by a mass flow controller (Yamatake MPC 9500B; Azbil Corporation, Kanagawa, and Japan; Air, N_2_ 0.5 L/min). The primary stream, consisting of clean air, flowed continuously through the bee throughout the conditioning. The secondary stream switched from an odor cartridge (during CS presentation) to a clean air cartridge (during non-CS presentation) using a solenoid valve controller. The clean air cartridge was prepared similarly to the odor cartridge, but without the ant sample.

### 2.3. Conditioning of the PER

Each bee was placed in the conditioning arena for 30 s. The first 13 s were designated for acclimation to the arena. The CS was then presented for 4 s, followed by a 3 s presentation of the US, with a 1 s overlap [39]. Finally, the bee remained in the arena for an additional 11 s to maintain the anticipatory link to the US [39]. In this research, bees were excluded if they responded to the CS during the first trial, failed to exhibit a proboscis extension response to the US across all acquisition trials, or extended their proboscis in response to the conditioning arena before the CS presentation and continued throughout the conditioning period. Each time, a cohort of 20 bees was tested until the sample size met the statistical requirements, ranging from 25 to 50, depending on the experiment.

### 2.4. Experiment 1: Learning Performance with Deceased Ant Odors

The associative learning performance of honeybees was examined using odors from three worker subcastes and female alates. Each odor sample weighed 0.037 g, representing 100 minors, 21 medians, 16 majors, and 6 female alates.

Paired conditioning: Bees underwent 5 acquisition trials with a CS–US pairing, followed by 5 extinction trials without the US. A cohort of 20 bees was tested with a 10 min inter-trial interval (ITI) (Figure S1A). Bees that met any of the criteria outlined in Section 2.3 were excluded from the experiment. The final sample size for the analysis ranged from 56 to 64 bees for each conditioning group.

Unpaired conditioning: To confirm that the responses were due to CS–US pairing, explicit unpaired conditioning was conducted alongside paired conditioning for 5 acquisition trials [40]. A cohort of 20 bees underwent a 5 min ITI. Half were exposed to stimuli in a pseudorandomized order, as follows: CS-US-US-CS-US-CS-CS-US-CS-US. The other half were exposed to stimuli in the following order: US-CS-CS-US-CS-US-US-CS-US-CS [39] (Figure S1B). Bees that failed to exhibit a proboscis extension response to the US across all trials were excluded. The final sample size for the analysis ranged from 57 to 61 bees for each conditioning group.

### 2.5. Experiment 2: Response Levels to Deceased Ant Odor Intensity

The study assessed honeybee responses to different ant odor intensities, starting with a single ant and gradually increasing to the number used in *Experiment 1*. Bees underwent 5 acquisition trials with the CS–US pairing (Figure S2). The CS included a varying number of ants: 1, 25, and 100 minor workers; 1, 5, and 21 median workers; 1, 4, and 16 major workers; and 1, 3, and 6 female alates. Bees that met any of the criteria outlined in Section 2.3 were excluded from the experiment. The final sample size for the analysis ranged from 51 to 62 bees for each group.

### 2.6. Experiment 3: Generalization across Deceased Ant Odors

The study investigated olfactory generalization across various ant odors. In this research, odors from four ant groups that elicited comparable responses in *Experiment 2* were selected as conditioned odors (CS). Bees were subjected to sequential training and testing phases [41] (Figure S3). Bees were trained with each CS over 5 acquisition trials during the training phase. Those that met any of the criteria outlined in Section 2.3 and failed to respond to the CS across all trials were excluded and did not proceed to the testing phase. In the testing phase, bees were randomly exposed to all four odors without the US (Figure S3). Bees that failed to respond to any of the tested odors during this phase were excluded. The final sample size for the analysis ranged from 34 to 39 bees for each group.

### 2.7. Experiment 4: Recognition of Live Ants

This experiment evaluated the ability of honeybees to recognize live *S. invicta* after training with varying numbers of deceased minor ants. It included the following three phases: training; testing; and rechecking (Figure S4). During the training phase, bees were conditioned with each of 1, 5, or 25 deceased minors across 4 acquisition trials. Those that met any of the criteria outlined in Section 2.3 and failed to respond to the CS across all trials were excluded and did not proceed to the testing phase. In the testing phase, six odors of live ants (10 minors, 1 minor, 10 medians, 1 median, 1 major, and 1 female alate) were presented in randomized order. Bees that failed to respond to any of the tested odors during this phase were excluded. In the rechecking phase, bees were retested with the same number of deceased minors used in training to confirm consistent responses to the conditioned odor. Bees failing to exhibit proboscis extension responses were excluded. The final sample size for the analysis ranged from 42 to 54 bees for each group.

### 2.8. Experiment 5: Discrimination of Ant Odors

This study examined the ability of honeybees to discriminate *S. invicta* odors from other ant species using odor discrimination and latent inhibition procedures. For odor discrimination, bees were exposed to alternating odors of *S. invicta* and one of five other ant species: *S. geminata*, *P. dives*, *P. fervens*, *P. rabo*, and *N. yaeyamensis*. The *S. invicta* odor (CS+, 10 minors) was paired with a sucrose reward, while odors from other ants (CS−, 10 randomly sized ants) were not (Figure S5). The CS− odors served as controls with which to evaluate the ability to discriminate between rewarded and non-rewarded odors [36,41,42]. Bees underwent conditioning with five trials each of CS+ and CS− in the following sequence: CS+ CS− CS− CS+ CS− CS+ CS+ CS− CS+ CS−. Rather than evaluating responses after each trial, the analysis focused on the bees’ reactions at the end of the conditioning process, specifically during the final two trials (1 CS+ and 1 CS−). This approach helps to assess the effectiveness of conditioning after repeated exposure to the stimuli. The difference in response between CS+ and CS− was measured using the discrimination index (DI) [43], calculated for each bee using the following expression:

$$Discrimination\;Index\;\left(DI\right)=Response\;to\;{CS}_{\left(last\;trial\right)}^{+}-\;Response\;to\;{CS}_{\left(last\;trial\right)}^{-}$$

The mean DI ranged from −1 to 1: −1 if the bee only responded to the CS−, 0 if responses to both odors were equal, and 1 if it only responded to the CS+. Bees that met any of the criteria outlined in Section 2.3 were excluded from the experiment. The final sample size for the analysis ranged from 30 to 32 bees for each group.

Latent inhibition, in the context of proboscis extension response studies, refers to a learning phenomenon where bees exhibit a delayed or reduced response to a CS paired with a sucrose reward if they were previously exposed to the CS without reinforcement [42,44]. In this research, latent inhibition was assessed by evaluating bee responses to inhibitor odors presented in 10 trials without sucrose reinforcement, following the results of Chandra et al. [45], in which a measurable latent inhibition effect was demonstrated after 8 unreinforced presentations. Ant odors poorly distinguished from *S. invicta* were used as inhibitors to evaluate whether this procedure could improve the discrimination ability of bees. The experiment comprised the following three phases (Figure S6): in the inhibition phase, bees underwent 10 trials with the inhibitor odor (10 randomly sized ants) without sucrose reinforcement; in the training phase, they experienced 4 trials with *S. invicta* odor (10 minors) paired with sucrose. Bees that met any of the criteria outlined in Section 2.3 during the training phase were excluded from the experiment; finally, in the testing phase, bees were exposed to both *S. invicta* and inhibitor odor in a randomized order. Responses were compared to a non-inhibition experiment where the inhibition phase was omitted and only the training and testing phases were included (Figure S6). The final sample size for the analysis ranged from 24 to 27 bees for each group.

### 2.9. Statistical Analyses

Analyses were conducted and verified using IBM SPSS Statistics 26 software. In Experiment 1, the dynamics of both the acquisition and extinction trials were investigated using Cochran’s Q test. This involved comparing the percentages of responses across 10 conditioning trials within the same group of bees. The responses between paired and unpaired conditioning in each trial were compared using a chi-squared test (Figure S1). In Experiment 2, responses from five trials were summed and ranked for each individual bee, ranging from 0 to 4. The Kruskal–Wallis test (H) was utilized to compare the ranked scores of associative learning across the four ant odors at the same ant weight, as well as across different ant numbers within each odor sample. Multiple comparisons were adjusted using the Bonferroni correction (Figure S2). In Experiment 3, the responses to four ant odors in the final trial of the training phase were compared using a chi-squared test. Cochran’s Q test was used to compare the response percentages to different odor samples within the same group of bees during the testing phases (Figure S3). In Experiment 4, during the training phase, the responses to the three deceased minor odors in the final trial were compared using a chi-squared test. During the testing phase, a Z test was used to compare proportions of proboscis responses (1 and 0) to live ant odors among three groups of bees trained with distinct deceased minors. Each of the three groups was paired with another group and tested accordingly (Figure S4). In Experiment 5 on discrimination, we assessed whether the bees had learned to differentiate between the odor of *S. invicta* (CS+) and the odor of another ant species (CS−). The responses to the CS+ and CS− from trials 3 to 10 for each individual bee were summed and ranked on a scale from 0 to 4. Each pair of CS+ and CS− was compared using the Wilcoxon signed-rank test. Responses from trials 1 and 2 were excluded because the first trial (CS+) was always 0, while the second trial (CS−) had responses. This exclusion allowed us to concentrate our analysis on trials that yielded relevant response data. The mean DI for each pairing of CS+ and CS− was tested for significant differences from zero using the Wilcoxon signed-rank test. The mean DI values among different ant species were compared using the Kruskal–Wallis (H) test. Multiple comparisons were adjusted using the Bonferroni correction (Figure S5). In Experiment 5 on latent inhibition, responses during the final trials of training phases for inhibition and non-inhibition procedures were compared using a chi-squared test. The responses between *S. invicta* and another ant odor during the testing phase were compared using the McNemar test (Figure S6).

## 3. Results

### 3.1. Experiment 1: Learning Performance with Deceased Ant Odors

**Figure 1:**
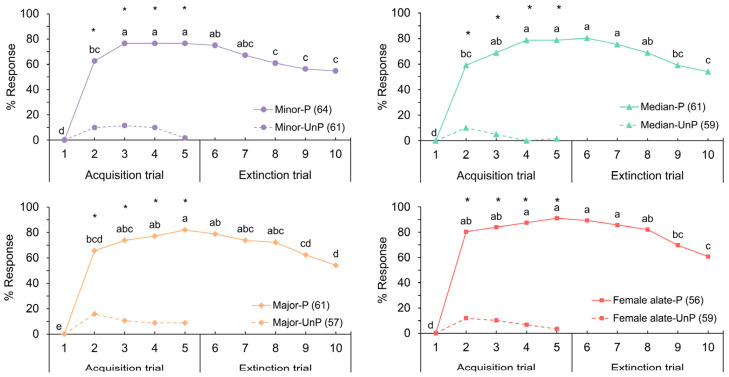
Percentages of responses to four deceased *S. invicta* odors during paired (P) and unpaired (UnP) conditioning (chi-squared test, * *p* < 0.001); letters denote significant differences among trials (Cochran’s Q test, *p* < 0.05). Numbers in parentheses indicate the sample size.

Honeybees exhibited the ability to detect and learn the odors emitted by all three deceased subcastes of worker and the deceased female alate of *S. invicta*. The conditioned responses of honeybees to all ant odors followed a consistent trend, transitioning similarly from the acquisition to the extinction trials (Figure 1). Honeybees quickly formed associations between ant odors and the sucrose reward. Throughout the trials, response levels remained significantly higher than those observed during the initial trial (Cochran’s Q test, df = 9, *p* < 0.05). Following a single CS–US pairing in all ant odors, 59% to 80% of bees exhibited the PER. The highest response levels were observed during trial 5, following four CS–US pairings (Cochran’s Q test, df = 9, *p* < 0.05), with response rates ranging from 76% to 91% across all ant odors.

During the extinction trials where the US was no longer presented, the response levels towards all ant odors declined. Nonetheless, over 50% of the bees exhibited PER in response to the ant odor, even in the absence of a reward for five consecutive trials (trials 10). The response levels between paired and unpaired conditioning (Figure 1) showed explicit heterogeneity from trial 2 to trial 5 across all ant odors (chi-squared test, df = 1, *p* < 0.001). These findings indicated that the performance observed in the paired conditioning resulted from associative learning.

### 3.2. Experiment 2: Response Levels to Deceased Ant Odor Intensity

**Figure 2:**
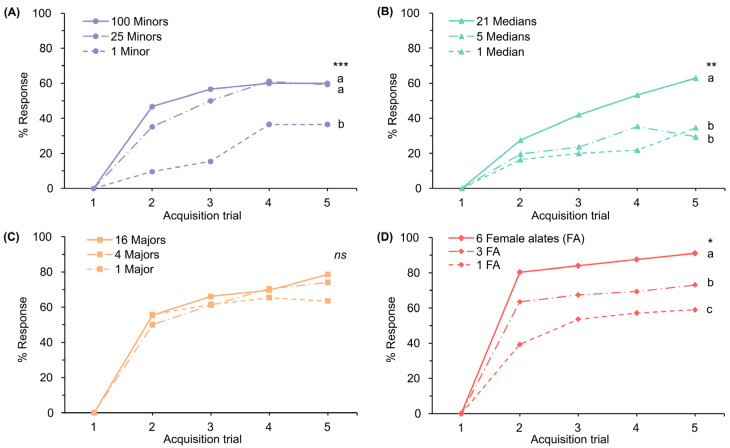
Percentages of responses during 5 acquisition trials to varying numbers of deceased *S. invicta*: (**A**) minors; (**B**) medians; (**C**) majors; and (**D**) female alates. Letters and asterisks denote a significant difference among groups (Kruskal–Wallis test, * *p* < 0.05, ** *p* < 0.01, *** *p* < 0.001, *ns*: not significant).

The responses of honeybees to the odors of deceased minor, median, major, and female alate ants of the same weight (0.037 g) revealed different ranks among these odors throughout the acquisition trials (Figure S7; Kruskal–Wallis test, H = 11.186, *p* < 0.05). We then investigated the responses to varying ant numbers. The results showed that the response levels to odor improved as the number of ants increased. In the minor workers, the response to a single ant was significantly lower than the response triggered by 25 and 100 ants (Figure 2A; Kruskal–Wallis test, H = 21.433, *p* < 0.001), with no significant difference between the responses to 25 and 100 ants. In the median workers, the responses to 1 and 5 ants did not significantly differ but were significantly lower than the response to 21 ants (Figure 2B; Kruskal–Wallis test, H = 14.300, *p* < 0.01). Regarding the major workers, no significant difference was noted among responses to 1, 4, and 16 ants (Figure 2C; Kruskal–Wallis test, H = 0.671, *p* = 0.715). In female alates, the response to six alates exceeded the responses elicited by three and one alate, respectively (Figure 2D; Kruskal–Wallis test, H = 30.584, *p* < 0.05).

### 3.3. Experiment 3: Generalization across Deceased Ant Odors

In line with Experiment 2, the comparison of responses in trial 5 showed that the percentage of responses to one deceased female alate closely resembled one major, 21 medians, and 25 minors, all within the same response range of 58–63%, derived from the lowest intensity of each ant odor. These intensities were used to examine the generalization of responses among different ant odors.

**Figure 3:**
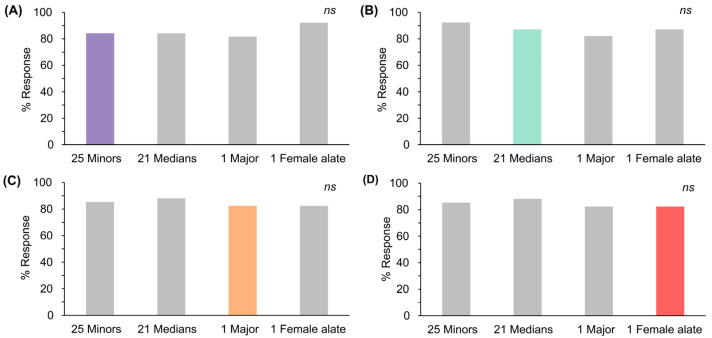
Percentages of responses to deceased *S. invicta* odors during the testing phase after conditioning with: (**A**) 25 minors; (**B**) 21 medians; (**C**) 1 major; and (**D**) 1 female alate. Colored bars indicate conditioned odors; grey bars indicate other odors (Cochran’s Q test, *p* > 0.05, *ns*: not significant).

During the training phase, responses in the final trial did not differ significantly across the selected deceased ant odors (Figure S8, chi-squared test, df = 3, *p* = 0.931). In the testing phase, responses after conditioning exhibited negligible differences between the various odors. After conditioning with the odors of 25 deceased minor and 21 median ants for 5 trials (Figure 3A,B; colored bars), responses to other ant odors exhibited variability, with some responses being either higher or lower than those elicited by the conditioned odor itself (Figure 3A,B; grey bars). Conversely, after conditioning with a single deceased major or female alate ant (Figure 3C,D; colored bars), responses to other ant odors were consistently higher than those elicited by the conditioned odors (Figure 3C,D; grey bars). Remarkably, the response patterns exhibited significant homogeneity across all conditioned ant odors. This indicated that bees exhibited high generalization in their responses among the odors of 25 minors, 21 medians, 1 major, and 1 female alate.

### 3.4. Experiment 4: Recognition of Live Ants

**Figure 4:**
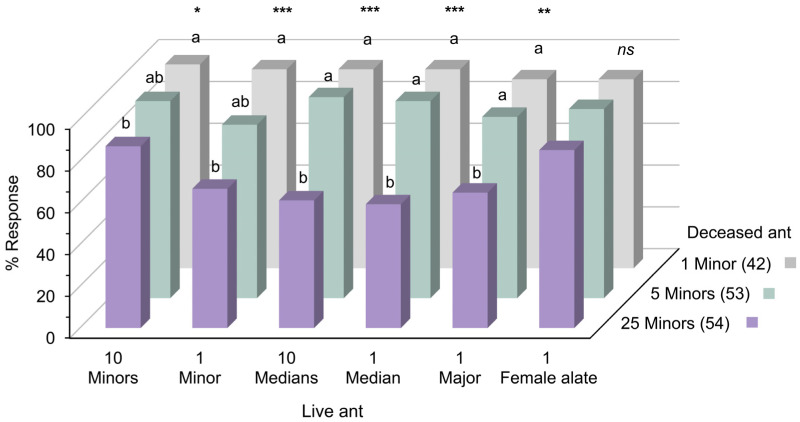
Percentages of responses to live *S. invicta* odors during testing phase, after conditioning to 1, 5, and 25 deceased minors. Letters and asterisks denote significant differences among groups (*Z* test, * *p* < 0.05, ** *p* < 0.01, *** *p* < 0.001, *ns*: not significant). Numbers in parentheses indicate the sample size.

The results indicated that honeybees could be trained using deceased *S. invicta* to recognize live counterparts. Bees conditioned with deceased minor workers successfully recognized live workers across all subcastes, as well as live female alates (Figure 4). Those trained with fewer ants (one or five minors) exhibited robust responses to all live ant odors, especially when the number or size of the tested ants increased. Conversely, bees conditioned with a larger number of deceased workers (25 minors) displayed weaker responses to all live workers (Figure 4; *Z* test, *p* < 0.001, 0.01, and 0.05). In live female alate recognition, bees trained with the highest number of deceased minors (25 ants) showed no significant difference in response rates compared to those trained with fewer ants. Notably, in the training phase, there were no significant differences in responses to 1, 5, and 25 deceased minor ants during the final trial, prior to testing the bees with live ant odors in the testing phase (Figure S9, chi-squared test in trial 4, df = 2, *p* = 0.511).

### 3.5. Experiment 5: Discrimination of Ant Odors

**Figure 5:**
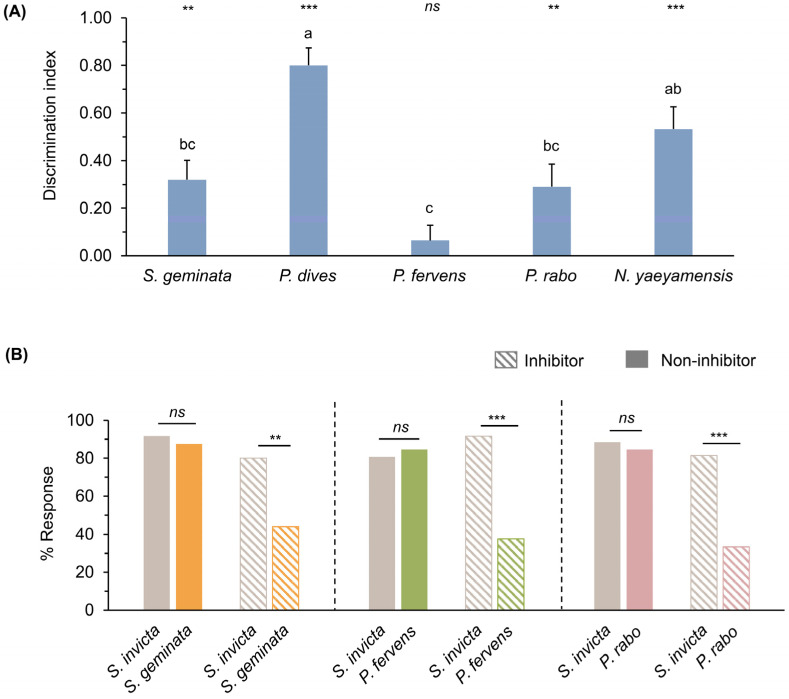
(**A**) Discrimination index during the final two trials comparing responses to *S. invicta* and five other ant species. Vertical bars represent the standard error. Letters indicate significant differences (Kruskal–Wallis test, H = 59.844, *p* < 0.01). Asterisks indicate significant differences from 0 (Wilcoxon signed-rank test, ** *p* < 0.01, *** *p* < 0.001, *ns*: not significant); and (**B**) percentages of responses to *S. invicta* versus three other ant species, with and without latent inhibition in the testing phase (striped and colored bars, respectively), McNemar test, ** *p* < 0.01, *** *p* < 0.001, *ns*: not significant.

Bee responses to *S. invicta* odors (CS+; paired with a sucrose reward) and other ant odors (CS−; no sucrose reward) from trials 3 to 10 varied across the different CS− odors (Figure S10). Responses to *S. invicta* odors were generally higher than those to other ant species, except for *P. fervens* (Figure S10, Wilcoxon signed-rank test, *p* = 0.813). Considering the discrimination index (DI), which reflects the bees’ ability to distinguish *S. invicta* from other ant odors (range: −1 to 1, with a median of 0), no significant difference was observed between the mean DI for *P. fervens* and 0 (Figure 5A; Wilcoxon signed-rank test, *p* = 0.371). In contrast, the DI for other ant odors significantly deviated from 0 (Figure 5A; Wilcoxon signed-rank test, *p* < 0.01 and 0.001). When comparing the mean DI across ant odors, bees exhibited the highest discrimination between *S. invicta* and *P. dives*, followed by *N. yaeyamensis* (Figure 5A; Kruskal–Wallis test, *p* < 0.01). In contrast, the ability to differentiate between *S. invicta* and *S. geminata*, *P. fervens*, and *P. rabo* was significantly lower (Figure 5A; Kruskal–Wallis test, *p* < 0.05).

Therefore, these three ant odors were subjected to additional testing using the latent inhibition procedure. They were presented to bees without sucrose reinforcement for 10 trials as inhibitor odors. The bees then underwent the training phase, and responses in trial 4 (the final trial) were compared between the latent inhibition and non-inhibition procedures, showing no significant difference between the two groups across the three inhibitor odors (Figure S11). The results during the testing phases showed that bees exhibited significantly lower responses to *S. geminata*, *P. fervens*, and *P. rabo* odors compared to *S. invicta* odors (Figure 5B; striped bars, McNemar test, *p* < 0.01 and 0.001). In contrast, without latent inhibition, the percentage of responses to the *S. invicta* odor and the three ant odors did not differ significantly (Figure 5B; colored bars, McNemar test, *p* > 0.05).

## 4. Discussion

This study established that pollen-foraging honeybees possess the ability to detect and respond to odors emitted by deceased *S. invicta*. Additionally, the bees demonstrated the capacity to be trained with deceased ants and subsequently recognize live ants. These findings suggest potential applications in regions where biosecurity regulations prohibit the transport of live *S. invicta* ants outside of infestation zones [12] or in areas that remain free from infestation. The use of deceased insects and their scent residues for training has also been successfully demonstrated in studies with dogs. These studies have utilized various stimuli, including towels scented with termites [46], filter paper infused with the odor of bed bugs [47], deceased stink bugs and their scent extracts [48], and, notably, filter paper absorbed with the scents of red imported fire ants [17].

### 4.1. Learning Performance with Deceased Ant Odors

The feasibility of using deceased *S. invicta* fire ants as odorants demonstrated that honeybees could be trained to detect and respond to odors from both deceased worker and female alates castes. The bees exhibited rapid learning, with extinction occurring when the association between odors and the sucrose reward was discontinued. Learning was further validated through parallel unpaired conditioning, which served as an experimental control.

### 4.2. Response Levels to Deceased Ant Odor Intensity

The intensity of odors significantly influences their effectiveness as olfactory stimuli [25]. Response rates are affected by stimulus concentration, with higher concentrations generally facilitating more rapid learning in associating the odor with a sucrose reward [25,37]. This pattern was evident in the transition rates from trial 1 to trial 2 across all ant odors, comparing responses to low versus high numbers of ants across all odors, as shown in Figure 2A–D.

Higher concentrations of conditioned stimuli can increase not only the response rates but also the response levels. This has been demonstrated by Suckling and Sagar [22], who observed increased response levels in bees with higher concentrations of tuberculosis signature compounds, and by Wright et al. [42], who found elevated response levels with an increased number of flowers used as conditioned stimuli. Our findings align with these studies, showing that an increase in the number of minors, medians, and female alate ants is associated with heightened honeybee response levels. In contrast, for major ant odors, bees exhibited strong responses even with a single ant, with no significant increase in responses when the number was raised to 4 or 16. This suggests that the odor from a single major ant may be sufficient to reach the detectability threshold for honeybees.

### 4.3. Generalization across Deceased Ant Odors

Given the uncertainty about the specific ant numbers and castes present in suspected objects or surveillance areas, a key question arises: which particular caste merits selection for training purposes? Can bees trained with any ant caste or subcaste detect others? To address this, our study evaluated the generalization of responses across three worker subcastes and female alate caste. The results indicated that, during the testing phase, bee responses exhibited insignificant differences between conditioned odors and other tested odors. Specifically, responses to tested odors were either higher, lower, or similar compared to those elicited by the conditioned odor. This contrasts with a study by Bonod et al. [41], which reported that the most conditioned odors induced the highest rate of responses. In our study, however, significant homogeneity was observed in responses between the conditioned odor and other tested odors, even when the conditioned odor varied among 25 minors, 21 medians, 1 major, or a female alate. This suggests that bees showed high generalization in their responses among the three worker subcastes and the female alate.

The high olfactory generalization can be attributed to the shared chemical composition of ant odors, which includes cuticular hydrocarbons [49], trail pheromones [50,51,52,53], and alarm pheromones [50,54]. Previous studies have shown that bees are more likely to generalize between odors with similar carbon chain lengths or within the same functional group [26,55]. In *S. invicta*, workers primarily produce *Z*,*E*-*α*-farnesene, along with a range of other terpenes [50], whereas female alates predominantly emit *β*-springene, along with *Z*,*E*-*α*-farnesene and additional terpenes [50]. The overlap in these terpene-based compounds likely contributes to the observed generalized responses in bees across these two different castes. Therefore, this study suggests that honeybees trained with the odor of one ant caste or subcaste can generalize their detection to include other castes or subcastes. This finding could enhance the training efficiency and improve the reliability of honeybee-based detection systems for *S. invicta*.

### 4.4. Recognition of Live Ants

To build on the finding that honeybees can learn to recognize and respond to deceased *S. invicta* odors, and given the potential applications in bio-detection systems, it is crucial to explore whether they can be trained with deceased ants to recognize live ants. This exploration is essential for developing practical applications in the real-world detection and surveillance of live *S. invicta*. In this experiment, we conditioned honeybees using the odors of deceased minor workers, which constitute the largest population in the colony and play a crucial role in foraging [30]. Our results showed that honeybees could be trained using deceased minor workers to recognize both live workers and female alates. Bees conditioned with lower-intensity odors of deceased minors exhibited stronger responses to live ant odors during the test phases than bees conditioned with higher-intensity odors. These findings are consistent with previous studies, which have shown that bees were more likely to generalize from low to high concentrations than from high to low concentrations [56,57]. Specifically, bees that associate a low concentration of an odor with a reward are likely to respond similarly to a higher concentration of the same odor. In contrast, if learning occurs at a high concentration, bees may not respond as strongly to the odor at a lower concentration. This pattern aligns with our results, where bees exhibited weaker responses to test odors when conditioned with the highest odor intensity (25 minor workers).

These results suggest that training honeybees with a single minor worker enables them to recognize all worker subcastes and female alate, whether tested with multiple ants or a single ant. Bees trained to recognize even a single ant are particularly suited for field detection needs in challenging environments such as international ports [1,10,11]. In these areas, *S. invicta* foraging activity may be minimal, with ants typically appearing as solitary workers or in groups of fewer than ten [11].

### 4.5. Discrimination of Ant Odors

Understanding the ability of honeybees to differentiate *S. invicta* odor from the other ant species is crucial for its application in detection and surveillance efforts, especially in areas where multiple ant species coexist with *S. invicta*. This capability would help in accurately identifying infestations and effectively targeting control measures. In this study, bees exhibited a high level of discrimination between the odor of *S. invicta* (CS+) and *P. dives* (CS−), followed by *N. yaeyamensis*. However, discrimination between *S. invicta* odor and the odors of *S. geminata*, *P. rabo*, and *P. fervens* was less pronounced. Notably, *Polyrhachis* and *Nylanderia* belong to the Formicinae subfamily, while *Solenopsis* and *Pheidole* belong to the Myrmicinae subfamily; thus, it could be hypothesized that subfamily-specific odors may influence discrimination levels. However, current data on cuticular hydrocarbons (CHCs) indicate that they are not reliable indicators for distinguishing ant subfamilies due to the variability in CHC profiles [58,59,60], which can be influenced by factors such as sex, caste, age, fertility, health status, and environmental conditions [60,61].

Given the observed difficulty honeybees faced in discriminating between the odors of *S. invicta* and other ant species, we implemented a latent inhibition procedure in which the specific ant odor was presented multiple times without reinforcement [42,44]. Remarkably, responses to the inhibitor ant odors (*S. geminata*, *P. rabo*, and *P. fervens*) were significantly lower compared to the responses to the *S. invicta* odor, even though these inhibitor odors were presented only 10 times, which was 4 times fewer than the number of presentations used in the study by Wright et al. [42]. This suggests that the application of latent inhibition can enhance bee ability to discriminate between ant odors.

Although honeybees demonstrated effective discrimination abilities in this study, further research is needed to explore alternative methods that may yield even better outcomes. For instance, pairing odors from other ant species (CS−) with an aversive stimulus, such as a salt solution [56] or a mild electric shock [27], might reduce responses to non-target ants and enhance discrimination ability, which could eliminate the need for a later application of inhibitor odors. If latent inhibition is required, increasing the number of inhibitor odor presentations may further decrease responses to that odor [45].

## 5. Conclusions

Our findings not only represent the first report of utilizing honeybees for detecting *S. invicta* odors but also for detecting insect odors in general. This study demonstrated that honeybees can detect and respond to odors from deceased *S. invicta* ants and recognize live ants, suggesting the potential for bio-detection in areas with strict regulations or no infestations. While bees showed rapid learning and high generalization across worker and female alate castes, challenges remain in discriminating between *S. invicta* and other ant species. Latent inhibition improved discrimination, but further research is needed to refine these methods. This approach could significantly contribute to the detection of invasive fire ants and other agricultural quarantine pests.

## Data Availability

The raw data supporting the conclusions of this article will be made available by the authors on request.

## Supplementary Materials

The following supporting information can be downloaded at: https://www.mdpi.com/article/10.3390/insects15100808/s1, Figure S1: schematic diagrams illustrating the experimental design and statistical tests used to assess learning performance with deceased ant odors: (A) the paired conditioning, pairing of CS and US in five acquisition and five extinction trials with a 10 min ITI; and (B) the unpaired conditioning, two pseudorandomized orders of ant odor-only (CS) and sucrose-only (US) presentations, each with a 5 min ITI. Figure S2: schematic diagrams illustrating the experimental design and statistical tests used to assess the response levels of honeybees to varying intensities of deceased ant odors. Figure S3: schematic diagrams illustrating the experimental design for the training and testing phases, along with the statistical tests used to assess olfactory generalization across different deceased ant odors. Figure S4: schematic diagrams illustrating the experimental design for the training, testing, and rechecking phases and statistical tests used to assess the study on recognition of live ants. Figure S5: schematic diagrams illustrating the experimental design and statistical tests used to assess the discrimination of ant odors. Figure S6: schematic diagrams illustrating the experimental design and statistical tests used to assess the latent inhibition procedure. Figure S7: percentage of responses to four deceased *S. invicta* odors, letters indicate a significant difference among odors across five trials (Kruskal–Wallis test; H = 11.186, Bonferroni correction; *p* < 0.05). Numbers in parentheses indicate the sample size. Figure S8: percentage of responses to four deceased *S. invicta* odors during the training phase of generalization procedure, chi-squared test in trial 5, *p* = 0.931, *ns*: not significant. Figure S9: percentage of responses to three deceased *S. invicta* odors in four trials during the training phases of live ants recognition procedure, chi-squared test in trial 4, *p* = 0.511, *ns*: not significant. Figure S10: percentage of responses to deceased *S. invicta* (CS+, the purple lines) and other ant species (CS−, the dotted lines) across five trials in the discrimination procedure (Wilcoxon signed-rank test from trial 3 to 10, **: *p* < 0.01, ***: *p* < 0.001, *ns*: not significant). Figure S11: percentage of responses to 10 deceased minors during the training phases of the latent inhibition procedure. The dotted lines represent the group of bees in the latent inhibition procedure, while the colored lines correspond to the bees in the non-inhibition procedure (chi-squared test in trial 4, *ns*: not significant).
