# Supplementary material for: Invasive Ant Detection: Evaluating Honeybee Learning and Discrimination Abilities for Detecting Solenopsis invicta Odor
Source: Insects. 2024 Oct 15;15(10):808. doi: 10.3390/insects15100808 (PMC11508661; doi:10.3390/insects15100808)
Supplement: Supplementary file 1 [file insects-15-00808-s001.zip › insects-3215979-supplementary.pdf]

Supplementary Materials

The experimental designs and statistical tests

Experiment 1: Learning performance with deceased ant odors

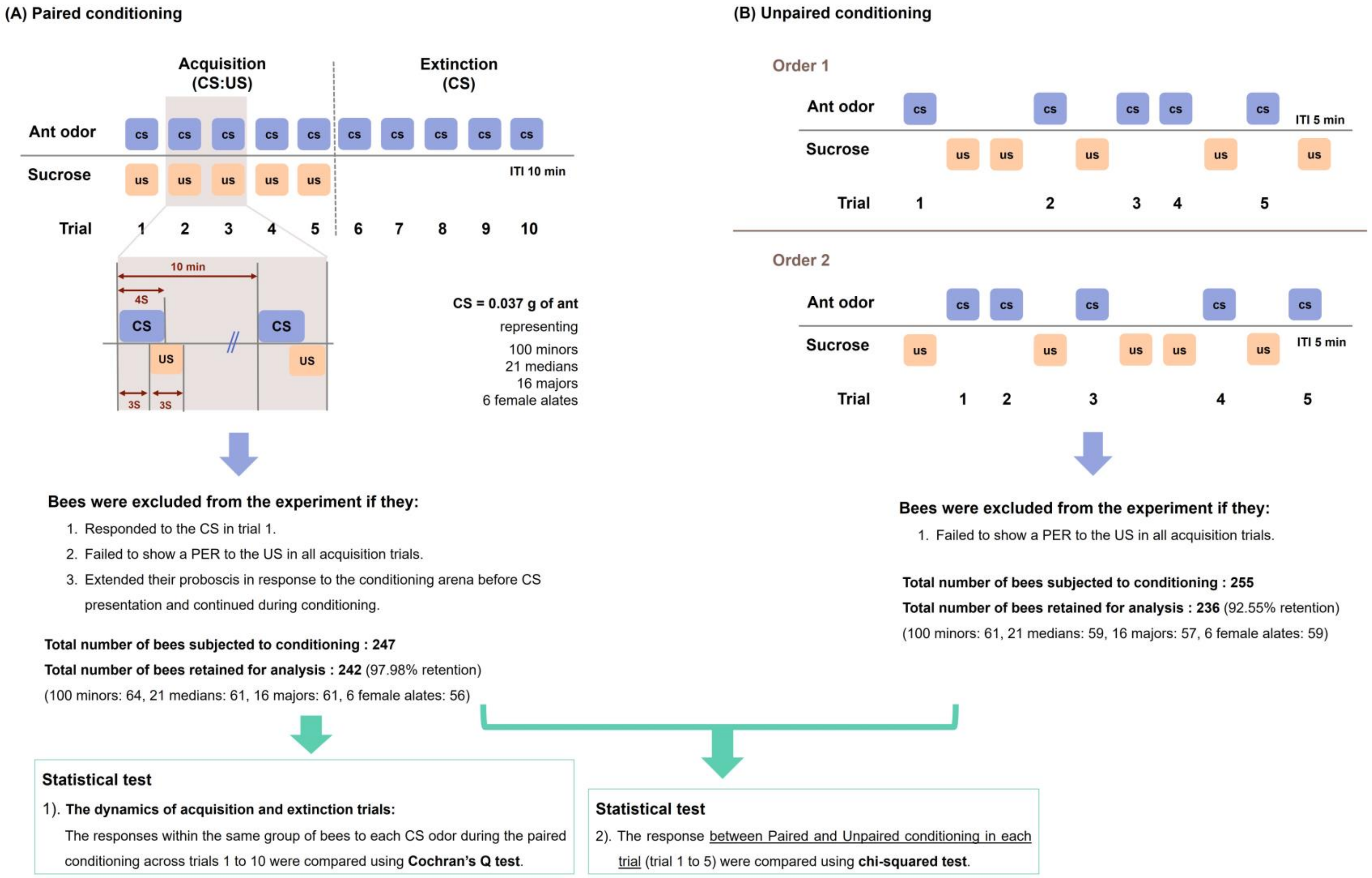

Figure S1. Schematic diagrams illustrating the experimental design and statistical tests used to assess learning performance with deceased ant odors. A) The paired conditioning, pairing of CS and US in 5 acquisition and 5 extinction trials with a 10-min ITI. B) The unpaired conditioning, 2 pseudorandomized orders of ant odor-only (CS) and sucrose-only (US) presentations, each with a 5-min ITI.

Experiment 2: Response levels to deceased ant odor intensity

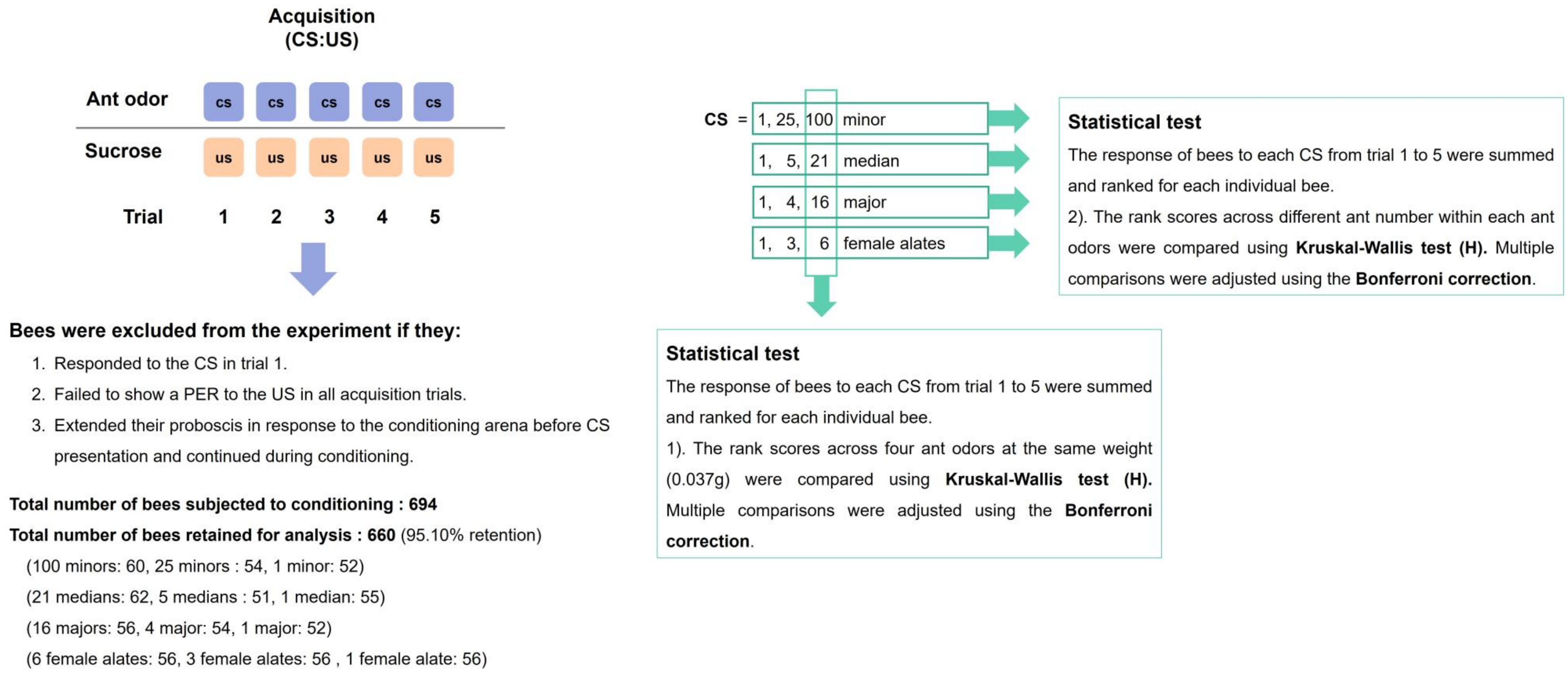

Figure S2. Schematic diagrams illustrating the experimental design and statistical tests used to assess the response levels of honey bees to varying intensities of deceased ant odors.

Experiment 3: Generalization across deceased ant odors

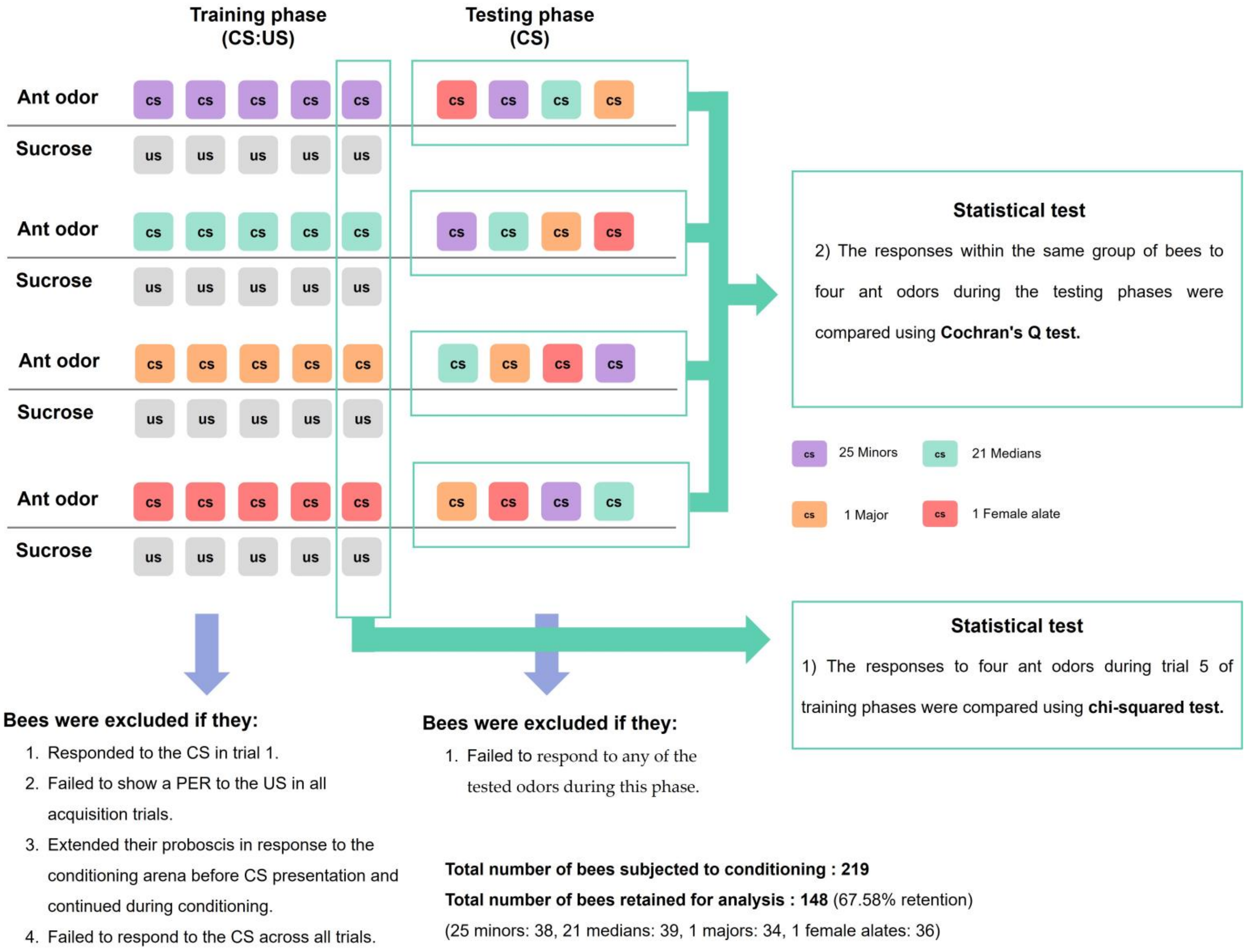

Figure S3. Schematic diagrams illustrating the experimental design for the training and testing phases, along with the statistical tests used to assess olfactory generalization across different deceased ant odors.

Experiment 4: Recognition of live ants

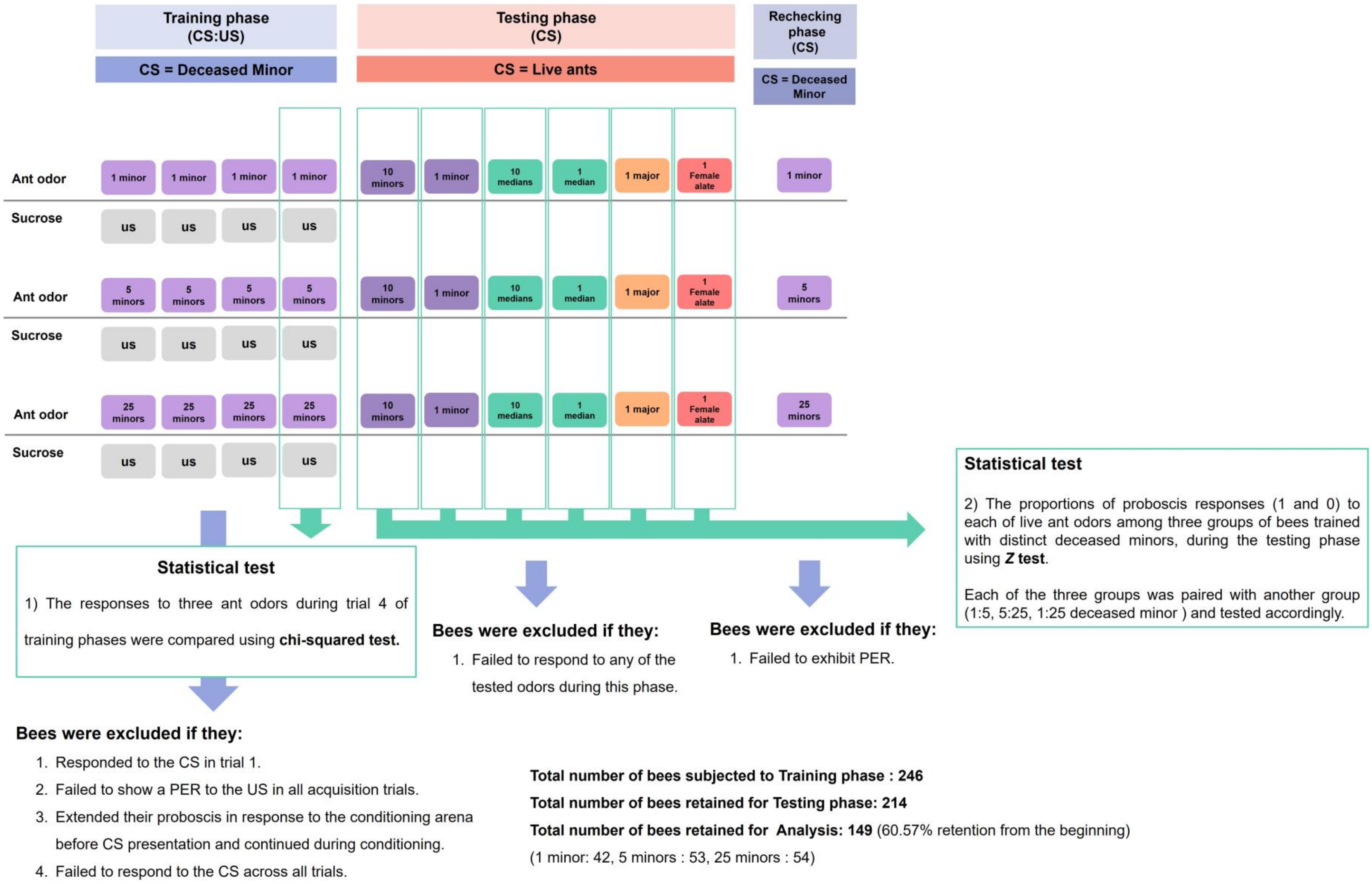

Figure S4. Schematic diagrams illustrating the experimental design for the training, testing, and rechecking phases and statistical tests used to assess the study on recognition of live ants.

Experiment 5: Discrimination of ant odors

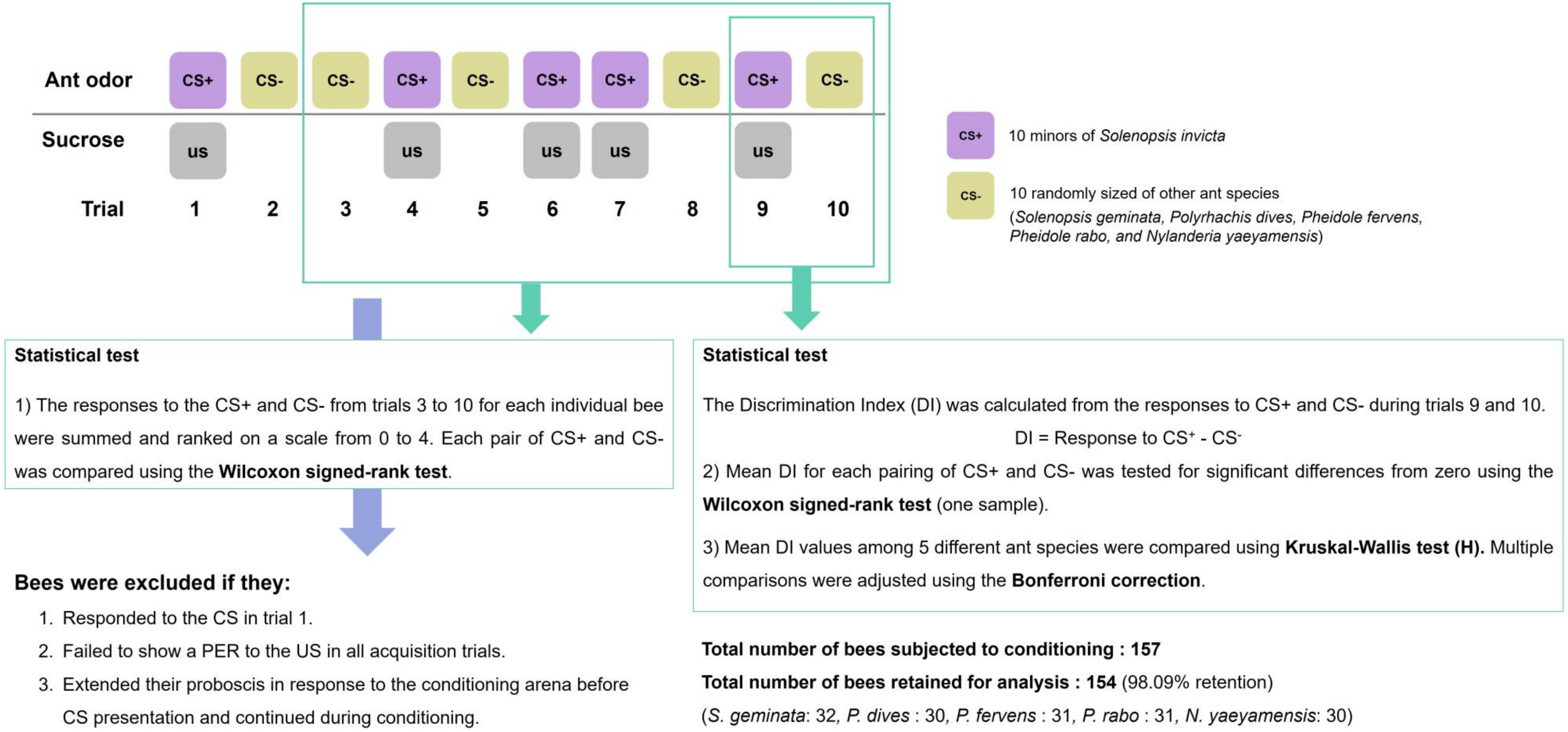

Figure S5. Schematic diagrams illustrating the experimental design and statistical tests used to assess the discrimination of ant odors.

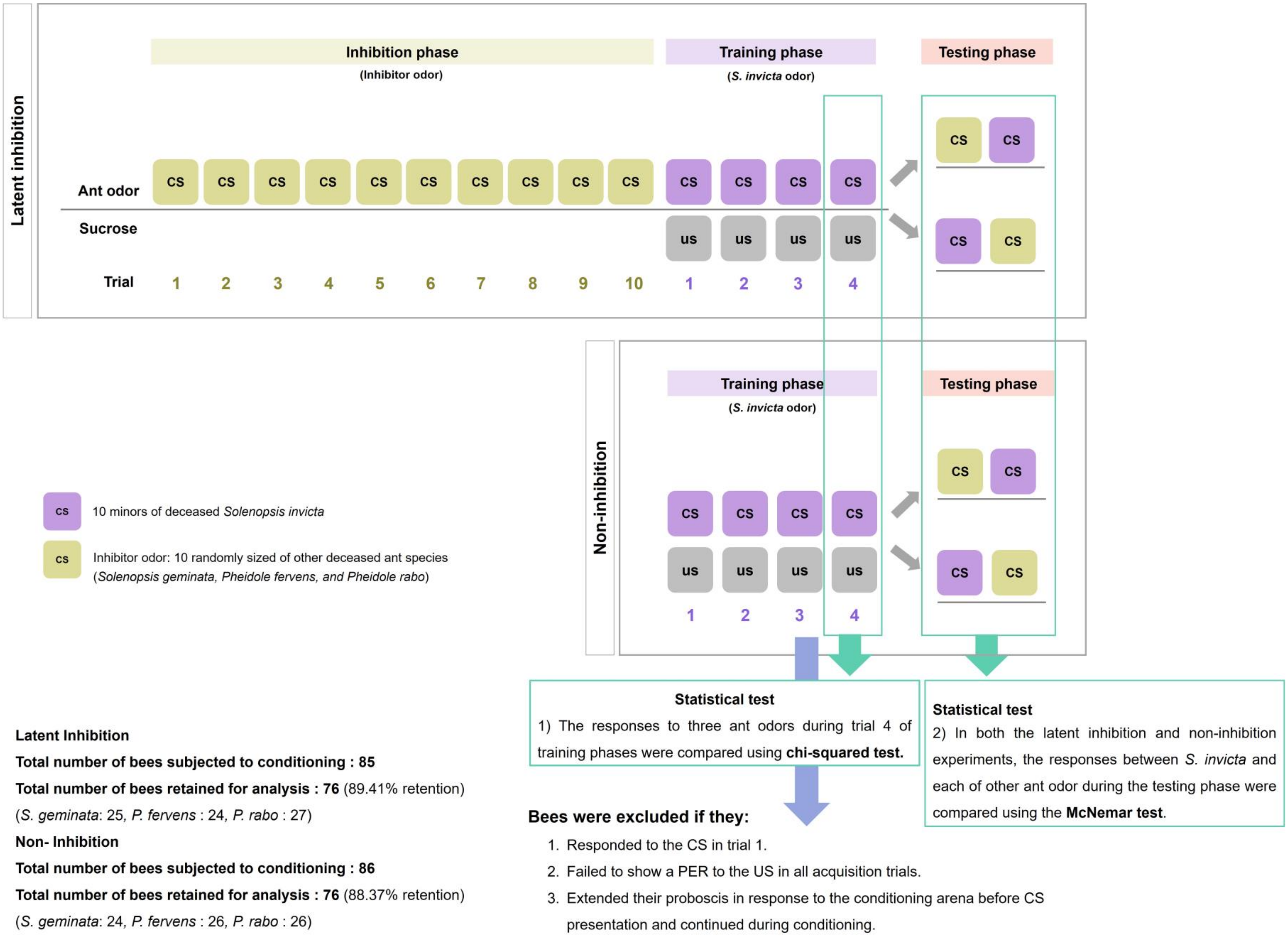

Figure S6. Schematic diagrams illustrating the experimental design and statistical tests used to assess the latent inhibition procedure.

The supplementary results

Experiment 2: Response levels to deceased ant odor intensity

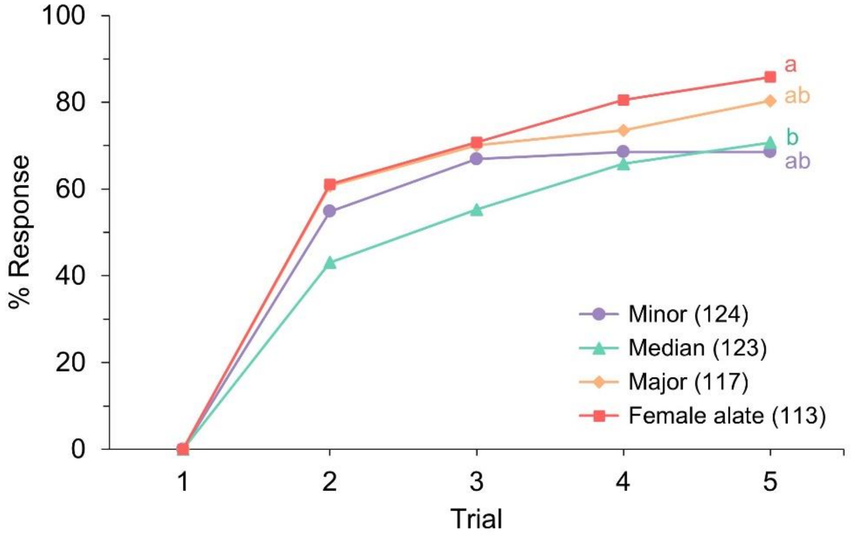

Figure S7 Percentage of responses to four deceased *S. invicta* odors, letters indicate a significant difference among odors across 5 trials (Kruskal-Wallis test; H = 11.186, Bonferroni correction; $p < 0.05$). Numbers in parentheses indicate the sample size.

Experiment 3: Generalization across deceased ant odors

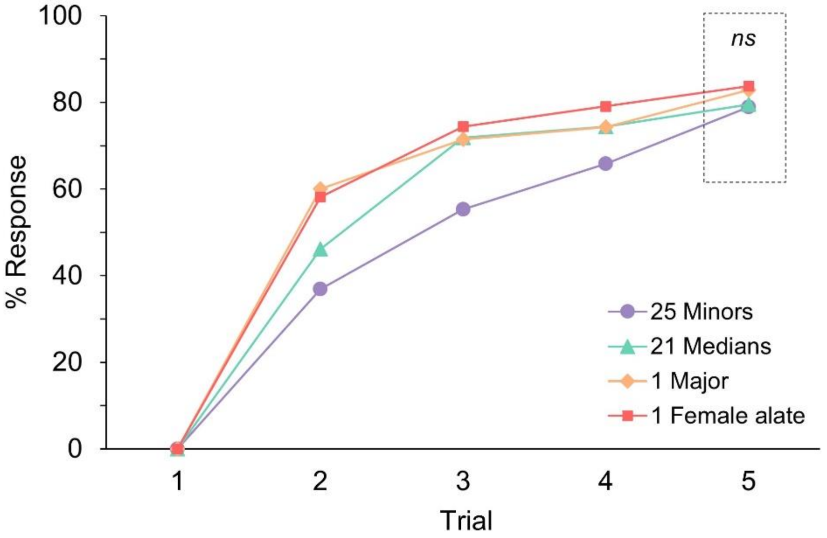

Figure S8 Percentage of responses to four deceased *S. invicta* odors during the training phase of generalization procedure, chi-squared test in trial 5, $p = 0.931$, *ns*: not significant.

Experiment 4: Recognition of live ants

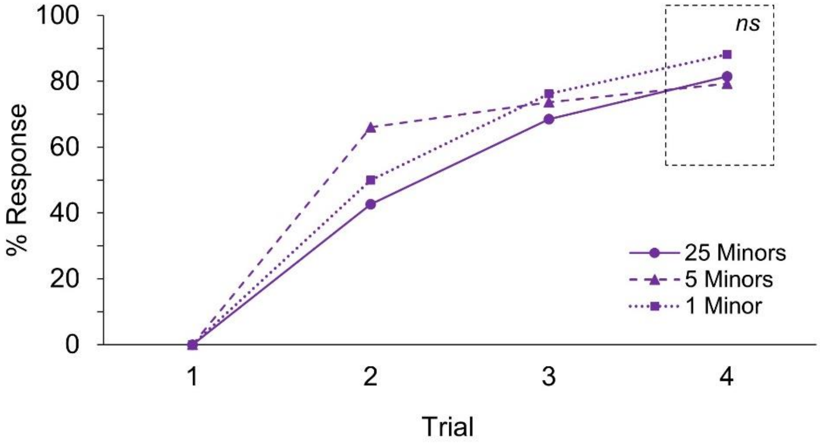

Figure S9 Percentage of responses to three deceased *S. invicta* odors in 4 trials during the training phases of live ants recognition procedure, chi-squared test in trial 4, $p = 0.511$, *ns*: not significant.

Experiment 5: Discrimination of ant odors

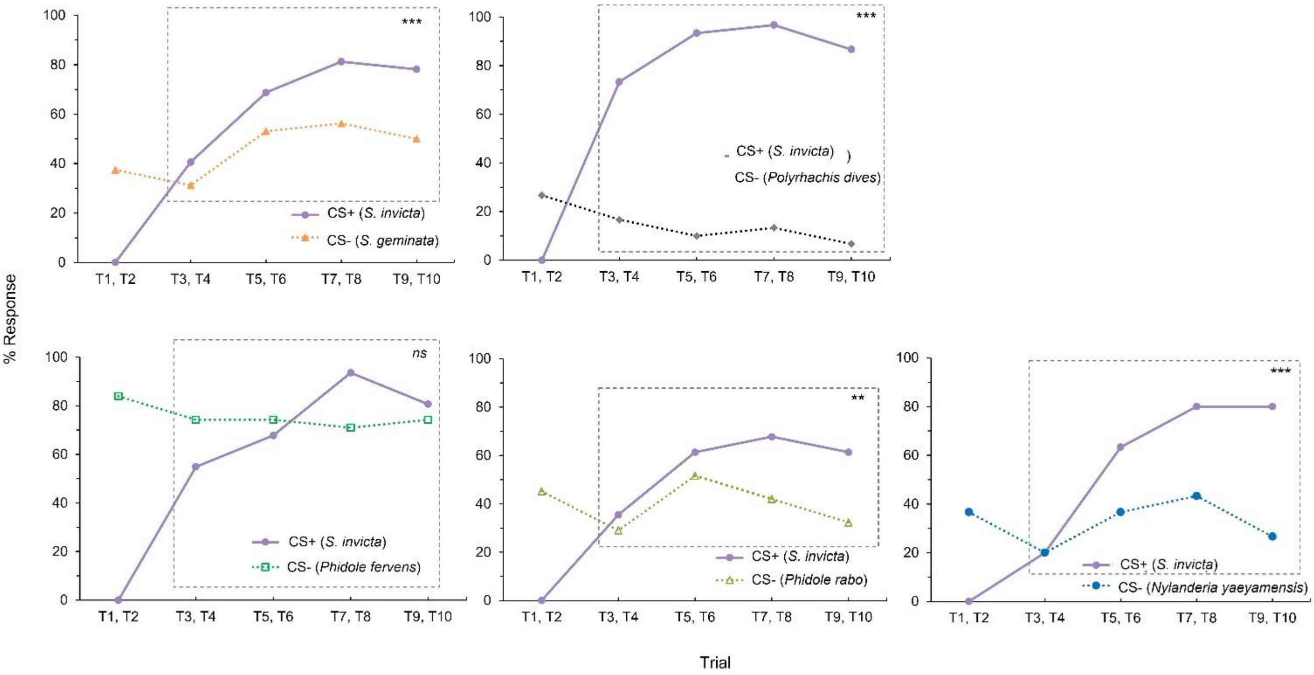

Figure S10 Percentage of responses to deceased *S. invicta* (CS+, the purple lines) and other ant species (CS-, the dotted lines) across 5 trials in the discrimination procedure (Wilcoxon signed-rank test from trial 3 to 10, **: $p < 0.01$, ***: $p < 0.001$, *ns*: not significant).

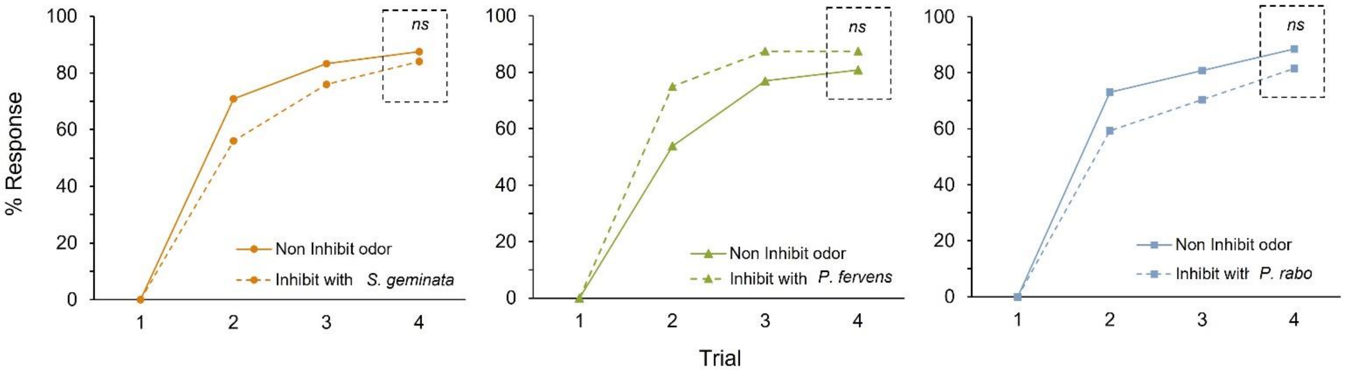

Figure S11 Percentage of responses to 10 deceased minors during the training phases of the latent inhibition procedure. The dotted lines represent the group of bees in the latent inhibition procedure, while the colored lines correspond to the bees in the non-inhibition procedure (chi-squared test in trial 4, *ns*: not significant).
